# Causal Structural Covariance Network Suggesting Structural Alterations Progression in Type 2 Diabetes Patients

**DOI:** 10.3389/fnhum.2022.936943

**Published:** 2022-07-15

**Authors:** Jiang Zhang, Yuyan Liu, Xiaonan Guo, Jing Guo, Zhengcong Du, Muyuan He, Qihong Liu, Dundi Xu, Taiyuan Liu, Junran Zhang, Huijuan Yuan, Meiyun Wang, Shasha Li

**Affiliations:** ^1^College of Electrical Engineering, Sichuan University, Chengdu, China; ^2^Med-X Center for Informatics, Sichuan University, Chengdu, China; ^3^School of Information Science and Engineering, Yanshan University, Qinhuangdao, China; ^4^Hebei Key Laboratory of Information Transmission and Signal Processing, Yanshan University, Qinhuangdao, China; ^5^MOE Key Lab for Neuroinformation, The Clinical Hospital of Chengdu Brain Science Institute, University of Electronic Science and Technology of China, Chengdu, China; ^6^School of Information Science and Technology, Xichang University, Xichang, China; ^7^College of Biomedical Engineering, Sichuan University, Chengdu, China; ^8^Department of Medical Imaging, Henan Provincial People's Hospital, Zhengzhou, China; ^9^Department of Endocrinology, Henan Provincial People's Hospital, Zhengzhou, China; ^10^The Athinoula A. Martinos Center for Biomedical Imaging, Department of Radiology, Massachusetts General Hospital, Boston, MA, United States; ^11^Harvard Medical School, Boston, MA, United States

**Keywords:** type 2 diabetes, voxel-based morphometry, gray matter volume, causal structural covariance network analysis, structural magnetic resonance imaging

## Abstract

**Background and Purpose:**

According to reports, type 2 diabetes (T2D) is a progressive disease. However, no known research has examined the progressive brain structural changes associated with T2D. The purpose of this study was to determine whether T2D patients exhibit progressive brain structural alterations and, if so, how the alterations progress.

**Materials and Methods:**

Structural magnetic resonance imaging scans were collected for 81 T2D patients and 48 sex-and age-matched healthy controls (HCs). Voxel-based morphometry (VBM) and causal structural covariance network (CaSCN) analyses were applied to investigate gray matter volume (GMV) alterations and the likely chronological processes underlying them in T2D. Two sample *t*-tests were performed to compare group differences, and the differences were corrected using Gaussian random field (GRF) correction (voxel-level *p* < 0.001, cluster-level *p* < 0.01).

**Results:**

Our findings demonstrated that GMV alterations progressed in T2D patients as disease duration increased. In the early stages of the disease, the right temporal pole of T2D patients had GMV atrophy. As the diseases duration prolonged, the limbic system, cerebellum, subcortical structures, parietal cortex, frontal cortex, and occipital cortex progressively exhibited GMV alterations. The patients also exhibited a GMV alterations sequence exerting from the right temporal pole to the limbic-cerebellum-striatal-cortical network areas.

**Conclusion:**

Our results indicate that the progressive GMV alterations of T2D patients manifested a limbic-cerebellum-striatal-cortical sequence. These findings may contribute to a better understanding of the progression and an improvement of current diagnosis and intervention strategies for T2D.

## Introduction

Diabetes is a disorder characterized by chronic high blood sugar level and four classification types: type 1 diabetes, type 2 diabetes (T2D), gestational diabetes, and other specific types of diabetes (Sharma and Shah, 2021). According to the International Diabetes Federation and World Health Organization, roughly 537 million adults (20–79 years) had diabetes in 2021, and the number is expected to increase in the foreseeable future (International Diabetes Federation., 2022). T2D accounted for over 95% of all diabetes cases (World Health Organization., 2021). Consequently, a study on T2D will benefit the majority of diabetic individuals and global health. It has been reported that T2D can cause cognitive function decline, including deficiencies in memory (De Sousa et al., 2021), attention (Marseglia et al., 2016), executive function (Oh et al., 2021), and visuospatial construction (Karvani et al., 2019); these changes are intimately related to brain alterations in brain structure (McCrimmon et al., 2012; Zhao et al., 2022).

T2D is associated with neuronal degeneration (Madhusudhanan et al., 2020; Yu et al., 2021), and a variety of neurodegenerative diseases are accompanied by gray matter (GM) abnormalities (Last et al., 2017). Previous studies have documented alterations in GM in T2D patients (Garcia-Casares et al., 2014; Liu et al., 2017; Redel et al., 2018; Roy et al., 2020; Feng et al., 2021). Roy et al. declared that T2D patients had gray matter volume (GMV) reductions in the prefrontal, hippocampus, amygdala, insular, cingulate, cerebellum, caudate, basal-forebrain, and thalamus compared with healthy controls (HCs) (Roy et al., 2020). Early-onset T2D patients had decreased GMV in the left orbital superior, middle, and inferior frontal gyri, as well as the right superior frontal gyrus, compared to HCs (Feng et al., 2021). Liu et al. considered cortical-striatal-limbic networks to be the main sites of GMV loss in T2D patients (Liu et al., 2017). Nonetheless, little published research has investigated whether progressive GM alterations exist in T2D and, if so, how the alterations proceed.

Voxel-based morphometry (VBM) analysis permits the whole-brain examination of structural changes or differences between groups at voxel levels (Kurth et al., 2015). Therefore, it is frequently used in studies of brain structural images. In this study, we intend to employ VBM analysis to determine the GMV of the subjects and thus quantify their brain structure. However, if we want to know the sequence in which structural changes occurred, we can use causal structural covariance network (CaSCN) analysis. CaSCN analysis originated from structural covariance networks (SCN) and Granger causality (GC) analyses. By analyzing correlations between the morphological properties of brain areas, SCN analysis can capture the patterns of covariation between distinct brain regions and may indicate the trajectories of development and maturity of brain regions (Alexander-Bloch et al., 2013; Yun et al., 2020; Prasad et al., 2022). Granger causality analysis can be used to describe the flow direction of information in different brain regions from time-series data and reflect the sequences of neural activities in these brain regions (Seth et al., 2015; Li et al., 2022). Combining SCN analysis and Granger causality analysis, CaSCN analysis calculates the GC values of structural changes in brain regions by artificially assigning time series to structural images (Zhang et al., 2017; Guo et al., 2020). GC values show how well the past values of one time series may predict the present values of another time series. This indicator is used to reflect the chronological order and likely causality of regional changes. Compared to SCN, CaSCN analysis can demonstrate the patterns of covariation of different brain regions and determine the chronological order of the structural changes in brain regions. In an effort to measure structural damage progression in temporal lobe epilepsy, CaSCN analysis was proposed, and the results revealed that the hippocampus, as a prominent node in the epileptic network, exerted causal effects on extrahippocampal regions (Zhang et al., 2017). The CaSCN analysis in patients with spinocerebellar ataxia type 3 (SCA3) showed progressive GM atrophy from the vermis to other regions in the cerebellum-neostriatum-cortical network (Guo et al., 2020). CaSCN analysis was also utilized to assess patients with generalized anxiety disorder (Chen et al., 2020) and autistic children (Guo et al., 2021). These results indicate that CaSCN analysis does allow for the investigation of possible disease progression sequences over a limited period. CaSCN analysis shapes pseudo time series and reveals the order of brain morphological changes. This has inspired us to use it to investigate the chronological order of structural alterations in patients with T2D.

Using VBM and CaSCN analyses, our study investigated GMV alterations and the GMV alterations chronological process in T2D patients over the course of their disease. In this study, an overall GMV comparative analysis was conducted to explore the whole brain GMV abnormalities of T2D patients. To further elucidate the progressive nature of the disease, GMV was compared between groups at different stages determined by illness duration. Subsequently, seed-based CaSCN analysis was performed to describe the possible progression of GMV abnormalities in T2D. Based on previous studies on brain morphologic changes in diabetes (Sato and Morishita, 2014; Roy et al., 2020), the following hypothesis was formulated: T2D patients would demonstrate abnormalities in GMV. As the duration of the disease progressed, GMV alterations in these regions would gradually change; allowing for the exploration of chronological order between regions.

## Materials and Methods

### Participants

All participants were recruited at Henan Provincial People's Hospital. Eighty-one T2D patients and 48 HCs were assessed in the investigation. Patients were diagnosed by the oral glucose tolerance test (OGTT), as suggested by the World Health Organization in 1999 (Canivell and Gomis, 2014), as well as other comprehensive examinations of glucose metabolism, fatty metabolism, and glycated hemoglobin (HbA1c). The inclusion criteria for HCs were as follows: (a) age and sex were similar to the disease group, and (b) the OGTT test examined people with normal glucose tolerance. The exclusion criteria for all subjects included depression or other neuropsychiatric diseases, diabetes mellitus complications (severe hypoglycemic coma; ketoacidosis, hyperosmolarity, and infection), disease or medical history causing central nervous system injury, drug or alcohol abuse, and systemic diseases. Table 1 presents the demographic information of all the subjects. All participants signed written informed consent in accordance with the Declaration of Helsinki. The Medical Ethics Committee of the Henan Provincial People's Hospital approved the study.

**Table 1:** Demographics clinical characteristics of the participants.

**Characteristics**	**T2D (*n* = 81)**	**HCs (*n* = 48)**	***P* value**
Age (years)	54.15 ± 9.26	54.13 ± 7.50	0.60^a^
Sex (male/female)	51/30	22/26	0.06^b^
Education			
Less than high school graduate	51 (63%)	10 (20.8%)	-
High school graduate	12 (14%)	5 (10.4%)	-
Some college or technical school	17 (21%)	20 (41.7%)	-
College graduate or more	1 (1%)	13 (27.1%)	-
Handedness (right/left)	81/0	48/0	-
Diabetes duration (years)	9.24 ± 6.61	-	-
Weight (kg)	70.5 ± 10.56	70.48 ± 10.36	0.96^a^
BMI (kg/m2)	24.93 ± 3.18	25.20 ± 10.92	0.62^a^
FPG (mmol/L)	9.30 ± 2.95)	-	-
HbA1c (%)	8.14 ± 1.71	-	-
MoCA score	26.96 ± 3.40	25.17 ± 2.08	<0.0001^a^

*Variables are represented as mean±standard deviation or values (percentage). T2D, Type 2 Diabetes; BMI, Body Mass Index; FPG, Fasting Plasma Glucose; HbA1c, Glycated hemoglobin; MoCA, Montreal Cognitive Assessment.*

*^a^Indicates P value for two-sample t-test.*

*^b^Indicates P value for X^2^ test*.

### Acquisition of Magnetic Resonance Imaging (MRI) Data

High-resolution three-dimensional T1-weighted structural images were acquired by a Siemens 3.0T Trio TIM MRI scanner at the Henan Provincial People's Hospital. A spoiled gradient recalled acquisition sequence was used during image collection with the following main scanning parameters: repetition time (TR), 1,900 ms; echo time (TE), 2.52 ms; flip angle, 9°; slice thickness, 1 mm; field of view, 250 × 250 mm; acquisition matrix, 256 × 256; voxel size, 0.97 × 0.97 × 1 mm; and slices, 160.

### Preprocessing

VBM analysis was performed on high-resolution T1-weighted images of all subjects using Data Processing Assistant for Resting-State fMRI (DPARSF, http://restfmri.net/forum/) and Statistical Parametric Mapping 12 (SPM12, https://www.fil.ion.ucl.ac.uk/spm/software/spm12/). The data preprocessing was performed on the DPARSF. First, the T1-weighted images were checked for artifacts and were manually reoriented to ensure that the origins were set at the anterior commissure. The reoriented images were subsequently normalized to Montreal Neurologic Institute (MNI) space; segmented into three different kinds of tissues named gray matter GM, white matter, and cerebrospinal fluid; and resampled to 1.5 × 1.5 × 1.5 mm^3^. Then, the images were modulated by using Jacobian determinants to acquire the GMV images. Finally, the resultant maps were smoothed by a Gaussian kernel filter with 6 mm full-width at half-maximum (FWHM).

### Statistical Analysis: Overall GMV Alterations in T2D

To demonstrate the overall GMV alterations in T2D, GMV data of T2D and HCs were compared using the two-sample *t*-test in SPM12. The mask used in the two-sample *t*-test was the optimal threshold mask, which was created with all subjects' GMV data (Ridgway et al., 2009). Age, sex, and total intracranial volume (TIV) were taken as covariates to remove their effects. Significant differences were corrected by Gaussian random field (GRF) correction (voxel-level *p* < 0.001, cluster-level *p* < 0.01).

### Statistical Analysis: Stage-Specific GMV Alterations Pattern in T2D

To investigate whether stage-specific characteristics and progressive changing patterns exist in T2D, we divided T2D patients and HCs into subgroups. We applied two grouping strategies: main and validation grouping strategies, to explore and verify the results.

In the main grouping strategy, T2D patients were grouped into two stages (stage 1: duration <9 years, stage 2: duration ≥ 9 years) in order of illness duration. We selected 9 years as the split since 9 is the median duration of our T2D patients. Consequently, we can divide the T2D patients into two roughly equal-numbered groups. Furthermore, this grouping strategy is similar to the long- and short-term duration division in a previous study on T2D (Huang et al., 2021). Additionally, age- and sex-matched control subgroups were created. To verify the results observed in the main grouping strategy, T2D patients were divided into three stages (stage 1: duration <6 years, stage 2: 6 years ≤ duration ≤ 11 years, and stage 3: duration >11 years) in the validation grouping strategy, and age- and sex-matched HCs subgroups were also created. This grouping strategy is similar to a previous T2D study (Thong et al., 2015).

A two-sample t-test with a GM optimal threshold mask and age, sex, and TIV as covariates was performed within each subgroup. The GRF correction (voxel-level *p* < 0.001, cluster-level *p* < 0.01) was conducted to test the differences within every subgroup. The regions where GMV alterations were observed in patients are displayed. Sex and age information for subgroups is shown in Table 2.

**Table 2:** Demographic and clinical variables in subgroups.

		**HCs**	**T2D**	***P* value**
Main grouping strategy	**Subgroup 1**			
	n	16	39	-
	Age (years)	50.44 ± 4.82	50.46 ± 7.47	0.99^a^
	Sex (male/female)	8/8	23/16	0.54^b^
	**Subgroup 2**			
	n	32	42	-
	Age (years)	55.97 ± 7.97	57.57 ± 9.52	0.44^a^
	Sex (male/female)	14/18	28/14	0.05^b^
Validation grouping strategy	**Subgroup 1**			
	n	10	28	-
	Age (years)	48.50 ± 4.81	48.39 ± 4.81	0.95^a^
	Sex (male/female)	5/5	17/11	0.56^b^
	**Subgroup 2**			
	n	21	27	-
	Age (years)	53.19 ± 5.91	53.63 ± 9.18	0.85^a^
	Sex (male/female)	9/12	18/11	0.18^b^
	**Subgroup 3**			
	n	17	26	-
	Age (years)	58.5 ± 8.14	60.88 ± 8.77	0.39^a^
	Sex (male/female)	8/9	16/10	0.35^b^

*Age is represented as mean±standard deviation.*

*^a^Indicates P value for two-sample t-test.*

*^b^Indicates P value for X^2^ test*.

### Seed-Based CaSCN Analysis

CaSCN analysis has recently been considered as a possible way to display GM alteration regions in temporal sequence in patients suffering from GM atrophy-related diseases (Zhang et al., 2017; Chen et al., 2020). Our analysis pipeline for GMV images of T2D subjects is very similar to that utilized in the aforementioned studies. The GMV files of T2D patients were sorted in ascending order of illness duration, thus constructing a pseudo time series for our cross-sectional data (Zhang et al., 2017; Guo et al., 2020). As the previous study did, the region showing the most significant GMV reduction (the right temporal pole with MNI coordinates: 32, 15, −26) was selected as the seed region for CaSCN analysis (Zhang et al., 2017). The duration of T2D has a cumulative effect on the brain (Saczynski et al., 2009), so the region with the most significant changes is likely to be the one affected by the disease initially. The average GMV values within the seed region (the right temporal pole) were extracted from the sequenced GMV maps and considered the pseudo-time series. The voxel-wise signed-path coefficient GC analysis was carried out in the REST (http://www.restfmri.net/forum/) toolkit. The equation (Zang et al., 2012) of signed-path coefficient GC analysis is as follows:


$$\begin{array}{l} {\;Y}_{t}=\overset{p}{\underset{k-1}{\sum }}A_{k}X_{\left(t-k\right)}+\overset{p}{\underset{i-1}{\sum }}B_{k}Y_{\left(t-k\right)}+{CZ}_{t}+\varepsilon_{t}\\ X_{t}=\overset{p}{\underset{k-1}{\sum }}{A^{\prime }}_{k}X_{\left(t-k\right)}+\overset{p}{\underset{i-1}{\sum }}{B^{\prime }}_{k}Y_{\left(t-k\right)}+{C^{\prime }Z}_{t}+\varepsilon_{t}^{{}^{\prime }} \end{array}$$

Where X_*t*_ and Y_*t*_ are the present values of the time series *X* and *Y*, *A*_*k*_ and $A_{k}^{{}^{\prime }}$ denote the signed-path coefficients, *B*_*k*_ and $B_{k}^{{}^{\prime }}$ represent the autoregression coefficients, *X*_*t*−*k*_ and *Y*_*t*−*k*_ are the past values of time series *X* and *Y*, *Z*_*t*_ is the covariate, and *C* or *C*′ are the coefficients of it, ε_*t*_ and $\varepsilon_{t}^{{}^{\prime }}$ are the residual errors. The output results are signed path coefficient maps.

To illustrate the GC influences the seed region had on whole-brain voxels, we focused on how well the seed region's past values could predict the present values of all voxels. Covariates include age, sex, TIV, and time interval of illness duration (the number obtained by subtracting the previous subject's illness duration from the latter subject after arranging all subjects in ascending order of illness duration). Then, the GC map was further Z-score transformed and GRF corrected (voxel-level *p* < 0.001, cluster-level *p* < 0.01).

## Results

### Overall GMV Alterations in T2D

T2D patients showed GMV atrophy in the limbic system (i.e., bilateral temporal pole extending to the parahippocampus, bilateral medial frontal gyrus extending to the superior frontal gyrus), bilateral supplementary motor area (SMA), visual cortex, bilateral insula extending to the superior temporal gyrus, bilateral precuneus, somatosensory cortex, and left inferior parietal lobule. And GMV increases were shown in the bilateral medial frontal gyrus, bilateral inferior frontal gyrus, bilateral superior temporal gyrus extending to the SMA, and middle occipital gyrus extending to the superior occipital gyrus. The results are shown in Supplementary Figure 1 and Supplementary Table 1.

### Stage-Specific GMV Alterations Pattern in T2D

**Figure 1:**
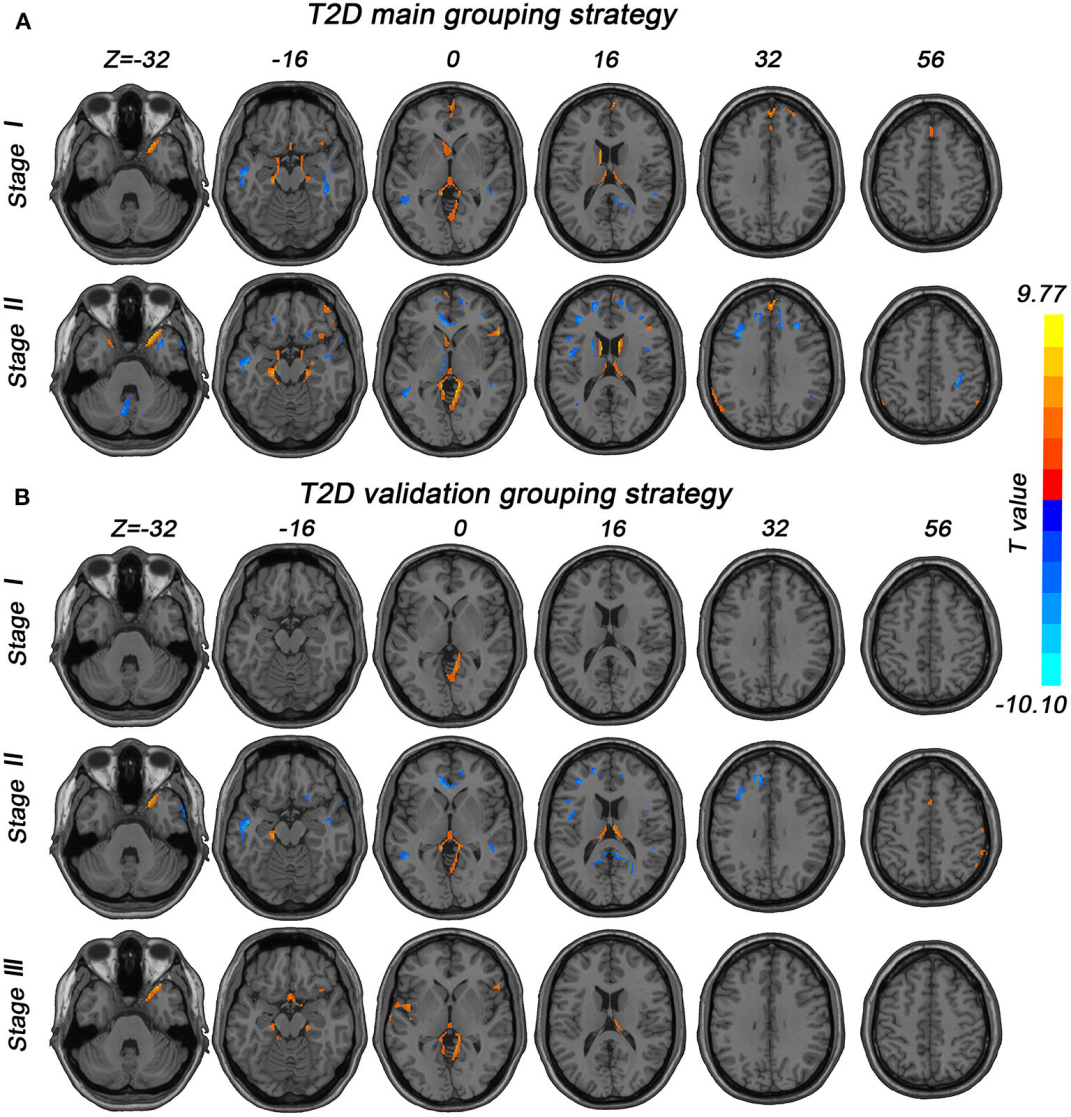
Stage-specific GMV alterations in T2D patients under different grouping strategies. GRF correction (voxel-level *p* < 0.001, cluster-level *p* < 0.01) was used in all subgroups. **(A)** Stages in the main grouping strategy were categorized by illness duration (stage 1, duration <9 years; stage 2, duration ≥9 years). **(B)** Stages in the validation grouping strategy were also divided by illness duration (stage 1, duration <6 years; stage 2, 6 ≤ duration ≤ 11 years; stage 3, duration >11 years).

In subgroup 1, GMV loss of T2D was observed in the right temporal pole, parahippocampal gyrus, prefrontal cortex, left caudate, bilateral thalamus, bilateral precuneus, and cerebellum, and GMV increase was observed in the language cortex. In subgroup 2, GMV reduction was located in the right caudate, bilateral inferior parietal lobule, left supramarginal gyrus, and the regions mentioned in subgroup 1. The GMV increase was mainly distributed in the language cortex, bilateral insula, right inferior parietal lobule, and prefrontal cortex. A validation grouping strategy was also conducted to verify our findings in the main grouping strategy. Figure 1 provides stage-specific GMV alterations in T2D under main and validation grouping strategies. The results of the two grouping strategies are very similar. Considering the results of the two strategies, generally speaking, GMV alterations in T2D begin in the right temporal pole, limbic system, and cerebellum, developing to subcortical structures, parietal cortex, frontal cortex, and occipital cortex.

### Seed-Based Causal Effects of GMV Alterations in T2D

The CaSCN analysis was believed to be able to depict the trajectory of disease by sequencing the morphometric data according to the illness duration of corresponding patients. We selected the most significant region showing GMV reduction in the overall two-sample *t*-test between patients and HCs as the seed region. We considered the GC effects from X (the seed region) to Y (voxels in the whole brain) (Jiang et al., 2018). Positive and negative GC values indicated the same or opposite GMV changes in Y, occurring after X (Jiang et al., 2018).

**Figure 2:**
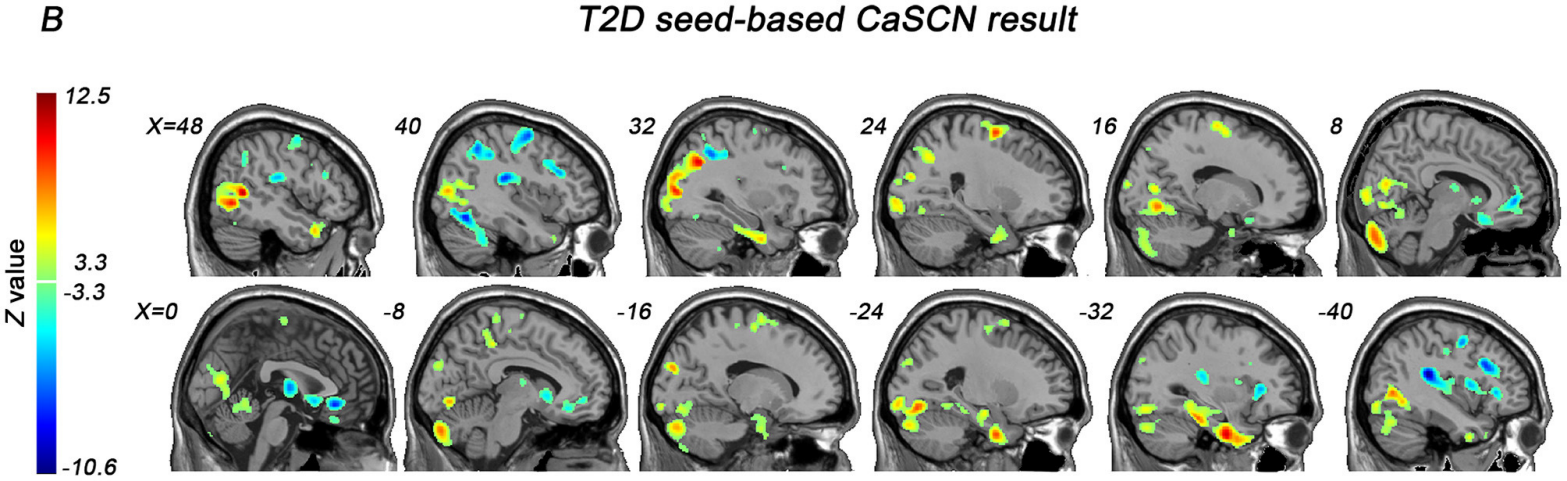
The right temporal pole-based CaSCN analysis results (GRF correction, voxel-level *p* < 0.001, cluster-level *p* < 0.01).

**Figure 3:**
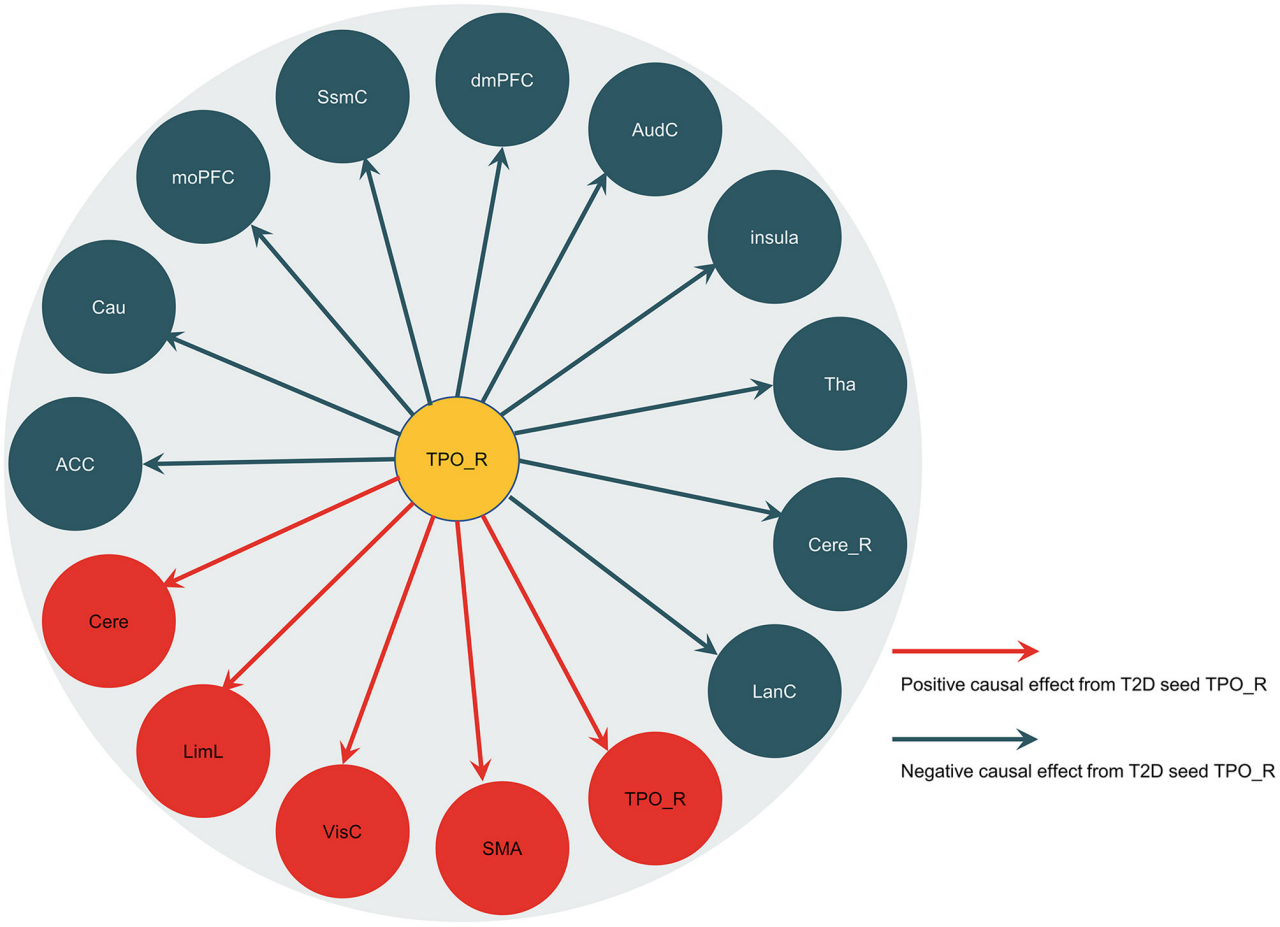
Summary of the right temporal pole-based CaSCN analysis results. The arrow lines represent the positive causal effects or the negative causal effects from the seed region (the right temporal pole). TPO_R, the right temporal pole; LimL, limbic lobe; VisC, visual cortex; SMA, supplementary motor area; LanC, language cortex; Cere_R, right cerebellum; Tha, thalamus; AudC, auditory cortex; dmPFC, dorsal medial prefrontal cortex; SsmC, sensorimotor cortex; moPFC, medial orbital prefrontal cortex; Cau, caudate; ACC, anterior cingulate cortex; Cere, bilateral cerebellum.

Figure 2 and Table 3 showed the results of the right temporal pole-based CaSCN analysis. A summary of the temporal pole-based CaSCN analysis results is presented in Figure 3. The seed region (the right temporal pole) of T2D patients exerted positive GC effects on the limbic lobe, SMA, visual cortex, and bilateral cerebellum (GRF correction, voxel-level *p* < 0.001, cluster-level *p* < 0.01). Negative GC values were observed in regions including the bilateral thalamus, bilateral caudate, anterior cingulate cortex (ACC), bilateral insula, auditory cortex, medial orbital prefrontal cortex (moPFC), dorsal medial prefrontal cortex (dmPFC), right cerebellum, language cortex, and sensorimotor cortex (GRF correction, voxel-level p <0.001, cluster-level p <0.01). Regions with positive and negative GC values had a decrease in GMV and an increase in GMV after GMV atrophy in the right temporal pole, respectively.

**Table 3:** The right temporal pole-based CaSCN analysis results.

**Brain regions**	**Hemisphere**	**MNI Coordinates** **(x, y, z)**	**GC value**	**Cluster size** **(voxels)**
Occipital lobe extending to temporal lobe and cerebellum	L/R	−43.5, −58.5, −9	9.45	9,558
Uncus extending to Parahippocampa Gyrus and FuG	L	−28.5, −4.5, −37.5	11.39	6,005
Uncus extending to parahippocampa Gyrus	R	30, −3.5, −37.5	6.5	1,080
Temporal pole	R	55.5, 6, −18	7.05	776
MTG extending to MOG and ITG	R	51, −54, 4.5	12.49	10,795
MFG extending to SFG and SMA	R	24, −3, 61.5	9.16	1,109
MFG extending to SFG and SMA	L	−19.5, −12, 61.5	6.34	838
FuG extending to cerebellum	R	37.5, −69, −18	−8.8	1,200
Caudate extending to medial frontal gyrus and ACC	L/R	0, 12, −6	−5.84	1,354
MFG extending to IFG and insula	L	−42, 21, 25.5	−7.87	2,884
Thalamus	L/R	0, −9, 6	−6.88	716
Insula extending to STG and IPL	L	−42, −36, 19	−10.51	3,691
Insula extending to PosG and STG	R	43, −25.5, 19.5	−7.86	1,344
dmPFC	R	37.5, 18, 28.5	−7.24	703
IPL	R	36, −54, 43.5	−9.26	1,582
PreG	L	−43.5, −3, 48	−7.08	685
MFG extending to PreG	R	40.5, −4.5, 58.5	−7.41	1,196
ACC extending to moPFC	L/R	1.5, 33, −9	−8.04	1,410
MFG extending to IFG and insula	L	−42, 21, 25.5	−7.87	2,884

*MTG, middle temporal gyrus; MOG, middle occipital gyrus; ITG, inferior temporal gyrus; MFG, middle frontal gyrus; SFG, superior frontal gyrus; SMA, supplementary motor area; FuG, Fusiform gyrus; ACC, anterior cingulate cortex; IFG, inferior frontal gyrus; STG, superior temporal gyrus; IPL, inferior parietal lobule; PosG, Postcentral gyrus; STG, superior temporal gyrus; dmPFC, dorsal medial prefrontal cortex; PreG, Precentral gyrus; moPFC, medial orbital prefrontal cortex*.

## Discussion

This study examined the stage-specific GMV alteration pattern and CaSCN of the right temporal pole in cross-sectional data of subjects with or without T2D. Compared with HCs, T2D patients demonstrated GMV abnormalities. The alterations follow a progressive pattern, targeting the limbic-cerebellum-striatal-cortical network. The CaSCN analysis revealed the temporal influences of the right temporal pole on other brain regions in T2D.

In the stage-specific GMV alterations investigation, a progressive GMV alteration pattern: limbic-cerebellum-striatal-cortical was revealed. In patients with T2D, we observed initial GMV alterations of the limbic system, including the right temporal pole, parahippocampal gyrus, and bilateral thalamus. With increasing disease duration, GMV atrophy spread to the cerebellum, and as the disease progressed further, GMV abnormalities progressed to the striatal and cortical cortex. Previous studies have identified abnormal limbic system connections in T2D, such as abnormal spontaneous brain activity (Peng et al., 2016) and mean diffusivity (Hsu et al., 2012). The early involvement of limbic system atrophy, similar to Alzheimer's disease (Scahill et al., 2002), suggests that T2D patients might be at risk for Alzheimer's disease. We found that the temporal pole of the limbic system, rather than the hippocampus (Gold et al., 2007), is the first brain region affected by T2D. The following reasons may explain this inconsistency: in the current study, we used data containing more subjects than in the previous study and found alterations located in other regions, instead of restricted to the hippocampus, in the early stage of T2D. The temporal pole is associated with some early stages of neurodegenerative disorders (such as amnestic mild cognitive disorders and Alzheimer's disease) and may account for the autobiographic memory deficiencies in these diseases (Herlin et al., 2021). Our finding of GMV atrophy in the temporal pole may provide tentative structural evidence of deficits in autobiographical memory in T2D patients (Li et al., 2021). Moreover, the right temporal pole is essential for emotional contagion and affective perspective-taking (Hillis, 2014). The increasing behavioral and psychological symptoms of T2D patients, such as apathy, may be traceable to right temporal pole abnormality (Sakurai et al., 2014; Sato and Morishita, 2014). GMV abnormalities in the temporal pole, as well as progressive GMV abnormalities, have been reported in first-episode SZ (Lee et al., 2016), a disease thought to be related to an increased risk for T2D (Mamakou et al., 2018). This is suggestive of a link between T2D and SZ. Given their shared role in emotion processing function (Wilcox et al., 2016; Pierce and Peron, 2020; Zhang et al., 2021b), it is possible that the cerebellum, which has been believed to be strongly connected with the limbic system (Schmahmann, 2000; Silvia et al., 2017; Zhang et al., 2021a), will proceed to develop GMV abnormalities after the limbic system. Poor emotion regulation has been observed in T2D patients (Rezaei et al., 2019; Pourmohammad Fahreh and Shirazi, 2020), and limbic-cerebellum GMV alterations might be the structural basis for this finding. Besides, the global Montreal Cognitive Assessment (MoCA) value (cognitive score) of T2D patients was positively correlated with the GMV atrophy in the cerebellum (Roy et al., 2020). Hence, the observed cerebellar GMV atrophy may have contributed to cognitive impairments in T2D patients. Altered functional connectivity between the cerebellar and cerebral networks in patients with T2D has been reported (Zhang et al., 2020). In addition, the cerebellum works in strict connection with the cerebral cortex and the basal ganglia (Milardi et al., 2019). This might suggest that the GMV abnormalities in the striatal-cortical network in later stages of T2D may be related to GMV abnormalities previously observed in the cerebellum. Smaller regional volume in the striatum of T2D patients was associated with higher glucose levels (Zhang et al., 2016). Observation of GMV alterations in striatal areas in later stages of T2D may indicate the significance of medical intervention for glucose control, particularly in the early stages of T2D.

Our CaSCN analysis results confirm that the right temporal pole was the first brain region affected by T2D. Specifically, the temporal pole exerts GC effects on the limbic system (ACC, medial orbital prefrontal cortex, insula, limbic lobe, the right temporal pole, and thalamus are mainly included in this study), cerebellum, striatum, and cerebral cortex. This means that atrophy of the right temporal pole precedes abnormalities in these regions. The right temporal pole exerts positive GC effects on the limbic lobe, SMA, visual cortex, and bilateral cerebellum. Our interpretation is that the GMV atrophy in the right temporal pole precedes and might have contributed to the atrophy in the aforementioned brain regions with positive GC values. Insulin was high in limbic regions, neocortex, and cerebellum (Blázquez et al., 2014). Therefore, the GMV atrophy in the right temporal pole and subsequent atrophy in the regions with positive GC values might suggest abnormalities in insulin-mediated neural pathways in T2D patients. Furthermore, the right temporal pole exerts negative GC effects on the bilateral thalamus, bilateral caudate, ACC, bilateral insula, auditory cortex, moPFC, dmPFC, right cerebellum, language cortex, and sensorimotor cortex. The thalamus has been deemed a significant relay to the auditory and sensorimotor cortex (Mitchell et al., 2014; Blumenfeld and Gummadavelli, 2018). The interaction between the cortico-basal ganglia-cortical circuit and regions of the prefrontal cortex is involved in behavior planning (Sandstrom et al., 2010). Previous studies have reported activation of the temporal pole, cerebellum, and basal ganglia during action planning and execution (Stegmayer et al., 2017; Errante and Fogassi, 2020). It can be thus speculated that the right temporal pole and the regions, mainly basal ganglia, cerebellum, prefrontal cortex, and sensorimotor cortex, jointly participate in motor planning and action execution. Atrophy of the right temporal pole may play a vital role in bringing out the compensatory GMV increases in these areas to maintain motor planning and executive function in patients with T2D. Furthermore, language processing is generally associated with strong left lateralization (Ardila et al., 2014; Cope et al., 2020). However, following the GMV atrophy in the right temporal pole, the GMV increase in the language cortex, which is not limited to the left hemisphere, might suggest that T2D patients have deficits in processing complex non-linguistic sounds (Cope et al., 2020).

### Limitations

The study was limited in several ways. First, it should be noted that the cross-sectional nature of this study determines that our findings do not directly reflect the sequence of structural alterations. These results are more referential and relative than absolute. Future longitudinal research on T2D patients could be an effective method for addressing this problem. Second, in addition to disease duration, the findings may have also been affected by diabetes's severity, treatment, comorbid conditions, and adequacy of control. Therefore, these factors should be considered and controlled in future research.

## Conclusion

In summary, our investigation of T2D indicated that the right temporal pole appeared to be the origin area affected by T2D. The progressive GMV abnormalities mainly followed a chronological order, targeting regions in the limbic-cerebellum-striatal-cortical network. Our work provides a clue to the likely GMV chronological process of T2D, which might shed light on disease diagnosis, facilitate early intervention, and reveal physiological and pathological mechanisms in T2D.

## Data Availability Statement

The raw data supporting the conclusions of this article will be made available by the authors, without undue reservation.

## Ethics Statement

The studies involving human participants were reviewed and approved by Medical Ethics Committee of the Henan Provincial People's Hospital. The patients/participants provided their written informed consent to participate in this study.

## Author Contributions

JiZ designed the research. JiZ, JG, and XG proposed the analysis method for processing the data. YL, JG, and XG analyzed the data. YL and XG prepared the figures. YL, XG, JiZ, JG, DX, and JuZ wrote the manuscript. TL, JuZ, HY, and MW acquired and interpreted the data. ZD, MH, QL, and SL provided support to this study during the experiments. All authors contributed to the article and approved the submitted version.

## Funding

This work was supported by the National Natural Science Foundation of China (Grant No. 61876114), the Med-X Center for Informatics funding project of Sichuan University (YGJC002), the 1·3·5 project for disciplines of excellence, West China Hospital, Sichuan University (ZYJC21041), and the Natural Science Foundation of Hebei Province (Grant No. H2021203002).

## Conflict of Interest

The authors declare that the research was conducted in the absence of any commercial or financial relationships that could be construed as a potential conflict of interest.

## Publisher's Note

All claims expressed in this article are solely those of the authors and do not necessarily represent those of their affiliated organizations, or those of the publisher, the editors and the reviewers. Any product that may be evaluated in this article, or claim that may be made by its manufacturer, is not guaranteed or endorsed by the publisher.
