# Supplementary material for: Causal Structural Covariance Network Suggesting Structural Alterations Progression in Type 2 Diabetes Patients
Source: Front Hum Neurosci. 2022 Jul 15;16:936943. doi: 10.3389/fnhum.2022.936943 (PMC9336220; doi:10.3389/fnhum.2022.936943)
Supplement: Supplementary file 1 [file Data_Sheet_1.PDF]

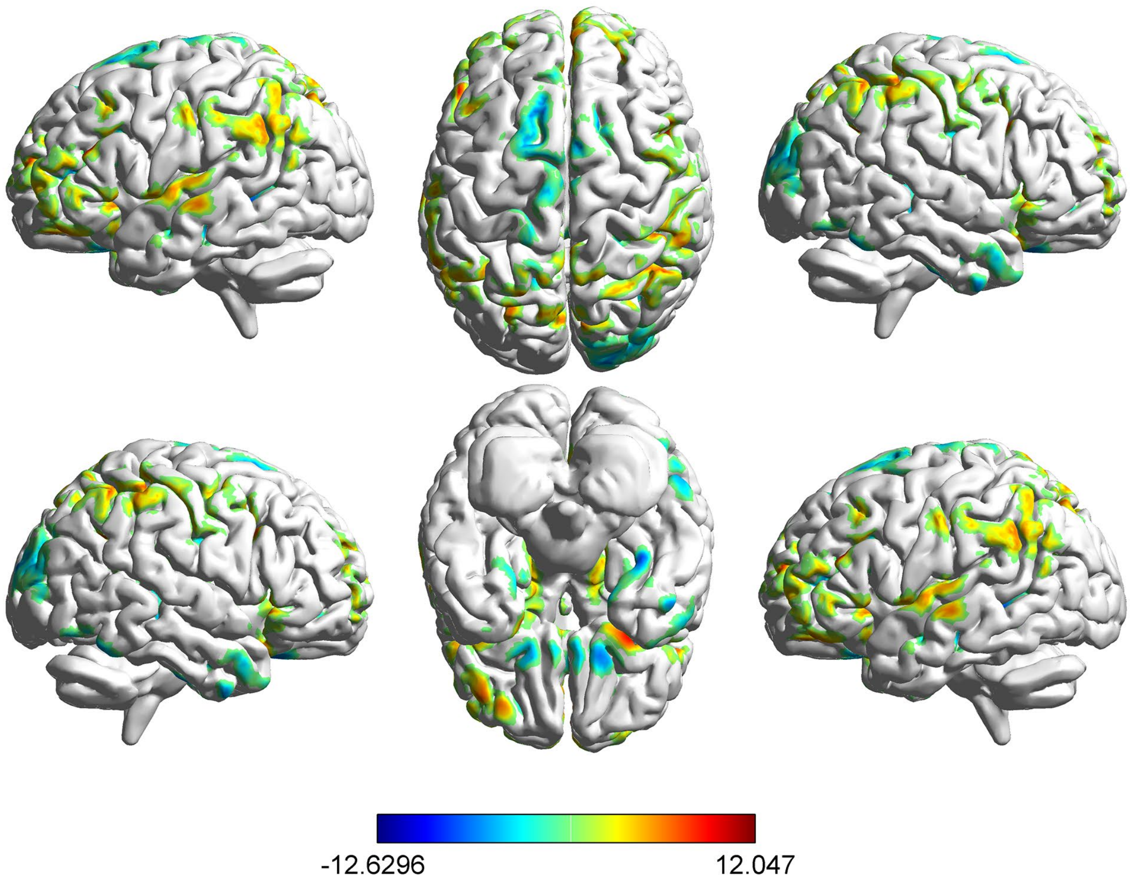

Supplementary Figure 1. Overall GMV alterations of T2D patients. Overall GMV alterations in patients with T2D. (GRF correction, voxel level p < 0.001, cluster level p < 0.01). The color bar represents t values.

Supplementary Table 1 Regions from the overall GMV alterations in T2D.

Brain regions	sphere	MNI Coordinates			Cluster size (voxels)
		x	y	z	
**HC>PAT**					
Limbic system	L/R	31.5	15	-25.5	11985
MFG extending to IFG	R	49	14	43	406
SMA	L/R	1.5	6	57	578
visual cortex	L/R	3	-78	40.5	850
insula extending to STG	L	-46.5	0	0	1313
precuneus	L/R	12	-60	70.5	752
somatosensory cortex	R	49.5	-54	54	1268
IPL	L	-43.5	-52.5	55.5	1023
insula	R	48	1.5	0	687
**PAT>HC**					
mFG	L/R	-12	48	12	498
IFG	L/R	42	25	7. 5	939
SFG extending to SMA	L/R	-14	19.5	64.5	1027
MOG extending to SOG	R	21	-97.5	19	693

MFG: middle frontal gyrus, IFG: inferior frontal gyrus; SMA: supplementary motor area, STG: superior temporal gyrus, IPL: inferior parietal lobule; mFG: medial frontal gyrus; SFG, superior frontal gyrus; MOG, middle occipital gyrus; SOG, superior occipital gyrus
